# Ecosystem change and human health: implementation economics and policy

**DOI:** 10.1098/rstb.2016.0130

**Published:** 2017-04-24

**Authors:** S. K. Pattanayak, R. A. Kramer, J. R. Vincent

**Affiliations:** 1 Sanford School of Public Policy, Duke University, Durham, NC, USA; 2 Nicholas School of the Environment, Duke University, Durham, NC, USA; 3 Duke Global Health Institute, Duke University, Durham, NC, USA

**Keywords:** conservation economics, impact evaluation, non-market valuation, cost–benefit analysis, policy analysis, implementation science

## Abstract

Several recent initiatives such as *Planetary Health*, *EcoHealth* and *One Health* claim that human health depends on flourishing natural ecosystems. However, little has been said about the operational and implementation challenges of health-oriented conservation actions on the ground. We contend that ecological–epidemiological research must be complemented by a form of implementation science that examines: (i) the links between specific conservation actions and the resulting ecological changes, and (ii) how this ecological change impacts human health and well-being, when human behaviours are considered. Drawing on the policy evaluation tradition in public economics, first, we present three examples of recent social science research on conservation interventions that affect human health. These examples are from low- and middle-income countries in the tropics and subtropics. Second, drawing on these examples, we present three propositions related to impact evaluation and non-market valuation that can help guide future multidisciplinary research on conservation and human health. Research guided by these propositions will allow stakeholders to determine how ecosystem-mediated strategies for health promotion compare with more conventional biomedical prevention and treatment strategies for safeguarding health.

This article is part of the themed issue ‘Conservation, biodiversity and infectious disease: scientific evidence and policy implications’.

## 1. Motivation

Does human health depend upon flourishing natural systems? A recent Lancet Commission discusses how environmental damage is hurting human health, especially for vulnerable sub-populations such as the poor, the socially disenfranchised, children and the elderly [1]. For example, declines in animal pollinators could exacerbate micronutrient deficiencies and non-communicable diseases. Similarly, deforestation and forest degradation in the tropics could increase infectious diseases such as diarrhoea, malaria and pneumonia, or at least make them harder to control.

To capitalize on perceived synergies between environmental conservation and public health efforts, the Lancet Commission proposes a new *Planetary Health* paradigm: ‘the achievement of the highest attainable standard of health, wellbeing, and equity worldwide through judicious attention to the human systems—political, economic, and social—that shape the future of humanity and the Earth's natural systems that define the safe environmental limits within which humanity can flourish’. This paradigm echoes themes from two other movements at the intersection of environmental conservation and public health. *EcoHealth* seeks to understand how social, economic and ecological factors and their interactions affect ecosystem ‘health’—the condition and sustainability of ecosystems—including the ability to provide ecosystem services, and the impact of these services on human health [2]. *One Health* is a worldwide strategy for expanding interdisciplinary collaborations and communications to address the health effects of interactions between humans, animals (both wild and domesticated) and the environment [3]. Each of these three initiatives reflects a growing concern that rapid and irreversible rates of environmental degradation will harm human health and well-being in ways that cannot be undone and ‘cured’ by medical treatments.

**Figure 1.**
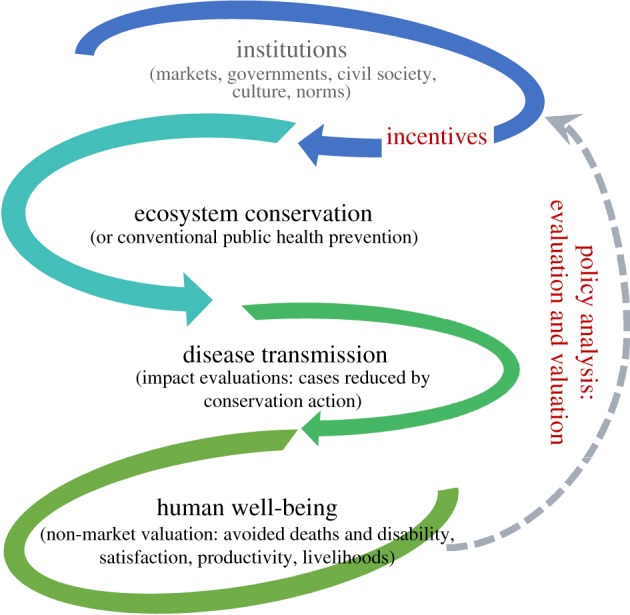
Policy analysis framework to evaluate how ecosystem conservation improves human health and well-being. As dotted arrow suggests, analyses can guide the design of appropriate incentives for conservation by using the long-term joint pay-offs—i.e. costs and benefits.

So far, Planetary Health, EcoHealth and *One Health* have said little about the operational and implementation challenges of health-oriented conservation actions on the ground. Their research foundations instead focus on elucidating the ecological–epidemiological mechanisms that connect ecosystem change to disease risks (which is part of our proposed framework). In this essay, we contend that these implementation challenges should provide the motivation for elucidating somewhat different, albeit coupled, sets of research questions on associated socio-economic mechanisms that are illustrated by figure 1 (adapted from [4]). That is, the eco-epidemiological research must be complemented by a form of implementation science that examines: (i) the links between specific conservation actions and the resulting ecological changes (second arrow), and (ii) how this ecological change impacts human health and well-being, when human behaviours are considered (third arrow).

Critically, such research can help us elucidate trade-offs implicit in pursuing conservation, development and health outcomes; projects and policies that ignored these trade-offs are counted as failures today [5]. Biologists will be familiar with the claim that even though genetic engineering could eliminate mosquitos that transmit diseases such as malaria, dengue or zika, the mosquito loss could negatively impact food chains, leave predators without prey and plants without pollinators [6]. Similarly, DDT was introduced to control pests of crops, improve agricultural yields and reduce poverty, but only after years of use, we understood that this also harmed birds and other species [7]. Thus, we are advocating for the policy evaluation tradition in public economics to identify and evaluate specific interventions that putatively deliver health-oriented ecosystem outcomes, when considering the full suite of benefits and costs. As shown in figure 1, such policy evaluations can thus feed institutional design and planning, including incentives (first arrow) to promote flourishing ecosystems where benefits exceed the costs (dotted arrow).

In the remainder of this short essay, we do two things. First, we present three examples of recent social science research on conservation interventions that affect human health. These examples refer to low- and middle-income countries in the tropics/subtropics, which is the context for the issues we consider in the essay. Each example illustrates only parts of the puzzle of determining policy implications. Unfortunately, the scientific literature on conservation and health is simply too thin—and thinner still on socio-economic aspects of this problem—to provide either sufficient nuance or a comprehensive picture of policies for ecosystem-mediated disease control [8]. Second, drawing on these examples, we present three propositions related to impact evaluation and non-market valuation that can help guide future research on conservation and human health. Research guided by these propositions will allow society to determine how ecosystem-mediated strategies for health promotion compare with more conventional biomedical treatments and cures for safeguarding health.

## 2. Illustrative cases

Much of the debate over conservation and health centres on the ‘dilution effect’: does increased biodiversity reduce or raise infectious disease risks [9,10]? Protected areas (PAs) are the oldest and best-known policy intervention for conserving tropical biodiversity, and they are also the most studied intervention, including by economists [11]. Our first case looks at PAs in the Brazilian Amazon. Although these PAs were established primarily to conserve biodiversity and slow deforestation and forest degradation, recent evidence suggests that they may have improved the health of local human populations. Bauch *et al*. [12] examined cases of malaria, diarrhoea and child pneumonia in all 750 *municípios* in the Brazilian Amazon from 2003 to 2006. They found that patterns of disease were correlated with the extent and type of forest conservation, specifically strict PAs, sustainable-use areas and indigenous territories. Controlling for weather, infrastructure, demographic and socio-political variables, and a variety of other confounders in a random effects panel model, the authors show that strict PAs reduced rates of all three diseases. Not all PAs were beneficial for health, however: sustainable-use PAs were positively correlated with malaria, but not the other two diseases. The difference in the effect on malaria is probably due to strict PAs effectively buffering human populations from exposure pathways, while sustainable-use areas do not. This case illustrates that blanket statements about the health impacts of tropical forest conservation are too simplistic. The type of conservation intervention matters, not only because it affects the amount and type of biodiversity, which is the focus of ecological–epidemiological research, but also because it influences human interaction with forests and the biodiversity they contain [13].

Since the 1990s, the perceived ineffectiveness of many PAs in low- and middle-income countries (paper parks) has prompted calls for devolving greater responsibility to local communities for forest conservation. Nepal is particularly well known for embracing decentralization and turning over the management and care of its forests to local community forest user groups (CFUGs). Against the backdrop of concern about climate change, and with support from NORAD, the government recently piloted a compensation scheme to incentivize CFUGs to enhance forest carbon stocks in the Terai, Hill and Mountain ecological zones of Nepal. The scheme is an example of a payment for ecosystem services (PES) programme [14] and is linked to the UN REDD+ programme, which aims at reducing carbon emissions from deforestation and forest degradation. Unlike the first case, the primary focus of Nepal's REDD+ programme is not biodiversity conservation. Nevertheless, might the programme influence patterns of infectious diseases? A study of 42 CFUGs, based on data from hundreds of households in each of the three ecological zones and using a panel-based difference-in-differences estimator, shows that the programme induced households to reduce use of biomass fuels and increase adoption of biogas digesters, a clean cooking technology that produces cooking gas from livestock dung [15]. These household behaviours are known to reduce household air pollution, the second ranked contributor to the global burden of disease due to its impact on respiratory infections and cardiovascular diseases. This case illustrates two points. First, forest conservation programmes are motivated by many concerns, not only biodiversity conservation, and examining the health impacts of only programmes of the latter type would risk overlooking important health impacts. Second, forest conservation programmes can have important health impacts that result not from changes in exposure pathways for zoonotic diseases endemic to forests but rather from changes in the use of forest resources (in this case, substituting away from use of firewood).

Carbon payment schemes like the REDD+ programme in Nepal might someday be the globally dominant form of PES, but watershed payment programmes are currently much more common in low- and middle-income countries [16]. Watershed PES programmes typically compensate landowners in upland regions for maintaining or enhancing forest cover. A long-held common rationale is that run-off from forestland is cleaner than run-off from other lands and an effective way to promote human health [17] or maybe even eliminating the need for water treatment, as the Catskill example suggests [18]. Waterborne infectious diseases are a major component of the global burden of disease; remote rural sites where treated water cannot be piped and water treatment cannot be established could benefit from watershed protection [4]. Recently, Vincent *et al*. [19] examined how operating expenses of municipal water treatment plants vary with upstream land uses in Malaysia. Using robust panel regression models with data from 41 water treatment plants during 1994–2007, the authors show that treatment costs are significantly lower for treatment plants downstream from forests compared to oil palm and rubber, with undisturbed (virgin) forests reducing costs substantially more than logged forests. Even for virgin forests, the study finds substantial variation in the size of the cost reduction. In addition to confirming that forests provide valuable water purification services, this case illustrates that those values can vary greatly even within relatively small distances, thus making it difficult to generalize about the impacts of conservation programmes. It also illustrates that conservation programmes can serve as complements instead of substitutes for conventional public health interventions, in this case by reducing the cost of supplying treated water. The case also highlights trade-offs implicit in promoting oil palm that has generated thousands of jobs and spurred economic development in Southeast Asia, while damaging health because of unanticipated air and water pollution [20].

## 3. Propositions

Health aspects of conservation are poorly understood [1,8]. The near-complete disconnect between the growing natural science research on the topic and the small body of associated social science research compounds the knowledge gap. Because conservation is operationalized and implemented in coupled socio-ecological systems, socio-politico-economic mechanisms become a critical part of the science–policy interface [21]. Incentives, institutions and human behaviour must be seriously considered; it is essential to understand why households, farmers, communities, companies, NGOs, donors and governments do what they do. Paying close attention to behaviour enables scholars and implementation scientists to: (i) measure impacts more accurately; (ii) develop a better understanding of preferences and constraints, which is necessary for characterizing market and non-market values (both costs and benefits); and (iii) identify policy levers that could trigger change to achieve social goals. We draw on an appraisal of the state of conservation policy science to offer the following propositions [11].

**Proposition 3.1.**

*While careful research on the linkages between diseases and ecosystem destruction and/or modification is necessary, it is not sufficient. Such work must be complemented by analysis of disease impacts of specific conservation interventions (the first two arrows in figure *1*), as highlighted in the previous section. It is now clear that these interventions are strategic, non-random and therefore a source for substantial selection bias in any statistical efforts to quantify the causal impacts of conservation* [11]*. By accounting for the strategic behaviours of various actors, we will be better placed to estimate the ‘delta health’ that is attributable to real life conservation actions (protected areas, integrated conservation and development projects, payments for ecosystem services, etc.) and support the development of decision support tools. While data for such evaluations are increasingly available from public sources, typically the health, environmental and policy data are collected and controlled by agencies and scholars who rarely cooperate with others working on different aspects of the problem, making data integration a formidable barrier.*

**Proposition 3.2.**

*Because conservation interventions will impose costs on society (including opportunity costs, as lands zoned for conservation could be deployed for other productive uses), it is imperative to compare the health benefits to these intervention costs and to compute a rate of return (the third arrow and links illustrated in figure 1). People's behavioural choices often signal how society values different outcomes, including health. Well-established non-market valuation protocols such as averting behaviour models, travel cost models, hedonic wage pricing and choice experiments have all evolved since the 1960s to derive social valuations of health and other policies* [13]*. Further, because conservation action will result in joint products, the societal benefits are typically multidimensional. As such, conservation could reduce specific disease clusters (*e.g. *waterborne diseases), but also trigger improvements in human well-being that are not related to health. If we are to give conservation a fair chance and consider the full suite of social benefits, we must use non-market and multi-criteria analysis tools to not just compare with the conservation costs but also to add health impacts (the delta health described in the previous proposition) to all the other benefits. Note, conservation actions will typically involve a full suite of direct costs and indirect opportunity costs (related to the trade-offs discussed previously). If the policy at hand impacts many economic sectors (say with PA expansion in Brazil), a computable general equilibrium approach is appropriate* [22]*. Broadly speaking, by studying patterns in human behaviours, we can derive the monetary valuations for these outcomes, which can be used to build an index of well-being and simultaneously value disparate outcomes such as bees, diarrhoea, malaria, oranges, orangutans, opportunity costs and programme expenses.*

**Proposition 3.3.**

*Since there is no uniformity in the landscape or context for either the ecological or the socio-economic processes underlying conservation, heterogeneity will underlie all of the impact evaluations or non-market valuations called for by the previous propositions. That is, looking at averages will be insufficient: particular pockets might be especially good for health-related conservation actions, while others are better for non-health ecosystem services, whereas yet other sites will represent situations where the opportunity costs simply outweigh any health or other benefits* [23]*. Perhaps the most straightforward way to address this is to repeat analyses for major sub-groups of people (poor, rich), places (remote, urban) and policies (regulatory, market-based), and consider realistic interactions of ecological and socio-economic drivers.*

Armed with a rate of return and/or cost–benefit estimate for conservation interventions, an analyst could compare such ecosystem-mediated health promotion efforts to conventional interventions for treating or preventing infectious diseases, such as immunizations, programmes for oral rehydration, zinc supplementation or insecticide-treated bed nets (as depicted in figure 1). Given the paucity of rigorous estimates of impacts (proposition 3.1), their values to society (proposition 3.2), and their distribution across space and social groups (proposition 3.3), however, it is simply too early to predict which interventions would win this horse race. However, two contextual considerations are paramount for a fair comparison. First, as a recent spate of criticism of experiments has documented [24], many impact estimates in public health come from highly controlled experiments with little or no recognition of the context that confronts poor, remote, disenfranchised households. Because this context does not vary in the experiment (or has been controlled), we do not know how effectiveness varies when contexts change. How effective are insecticide-treated bed nets in forest fringe or mining communities where conservation interventions are working, compared to locations where they are not? Second, presumably such a comparison could be brought to discussions with finance ministries, not only environmental or health ministries. Unlike a health ministry, a finance ministry might well ask (and appropriately so from a broad social welfare standpoint) if conventional health approaches deliver any of the environmental and other benefits associated with conservation instead of focusing on just health efficacy.

In closing, beyond the typical call for simply more research on ecosystem-mediated health outcomes, we are arguing for more of a particular kind of research—one that focuses on how more distal causes (such policy implementation and human behavioural adjustments) affect ecosystems and the consequences for human well-being, including health impacts. Such research follows an established tradition for comprehensive project appraisal that includes all social costs and benefits [25]. Naturally, this requires a multidisciplinary multi-method pluralistic strategy to understand not only how ecosystem modifications affect diseases, but just as critically, what we can do about the problem. Such research would be an appropriate response to recent calls in sustainability sciences and public health for more ‘strategic scholarship’ [26], use-defined contextual research [27,28] and ‘new social contracts’ between scholars and practitioners [21]. Otherwise, we risk perpetuating a type of ignorance trap in which there is little research on the impacts of conservation on health because there are few conservation-based health interventions to study, and there are few to study because evidence on their impacts is lacking.

## References

[1] Whitmee S et al. 2015 Safeguarding human health in the Anthropocene epoch: report of The Rockefeller Foundation–Lancet Commission on planetary health. Lancet 386, 1973–2028. (10.1016/S0140-6736(15)60901-1) 26188744

[2] Mi E, Mi E, Jeggo M 2016 Where to now for one health and ecohealth? EcoHealth 13, 12–17. (10.1007/s10393-016-1112-1) 26968555 PMC7088312

[3] Gibbs EP 2014 The evolution of one health: a decade of progress and challenges for the future. Vet. Rec. 174, 85–91. (10.1136/vr.g143) 24464377

[4] Pattanayak SK, Wendland KJ 2007 Nature's care: diarrhea, watershed protection, and biodiversity conservation in Flores, Indonesia. Biodivers. Conserv. 16, 2801–2819. (10.1007/s10531-007-9215-1)

[5] McShane TO et al. 2011 Hard choices: making trade-offs between biodiversity conservation and human well-being. Biol. Conserv. 144, 966–972. (10.1016/j.biocon.2010.04.038)

[6] Fang J 2010 Ecology: a world without mosquitoes. Nature 466, 432 (10.1038/466432a) 20651669

[7] Kinkela D 2011 DDT and the American century: global health, environmental politics, and the pesticide that changed the world. Chapel Hill, NC, USA: University of North Carolina Press.

[8] Myers SS, Gaffikin L, Golden CD, Ostfeld RS, Redford KH, Ricketts TH, Turner WR, Osofsky SA 2013 Human health impacts of ecosystem alteration. Proc. Natl Acad. Sci. USA 110, 18 753–18 760. (10.1073/pnas.1218656110) PMC3839693 24218556

[9] Civitello DJ et al. 2015 Biodiversity inhibits parasites: broad evidence for the dilution effect. Proc. Natl Acad. Sci. USA 112, 8667–8671. (10.1073/pnas.1506279112) 26069208 PMC4507196

[10] Salkeld DJ, Padgett KA, Jones JH 2013 A meta-analysis suggesting that the relationship between biodiversity and risk of zoonotic pathogen transmission is idiosyncratic. Ecol. Lett. 16, 679–686. (10.1111/ele.12101) 23489376 PMC7163739

[11] Ferraro PJ, Lawlor K, Mullan KL, Pattanayak SK 2011 Forest figures: ecosystem services valuation and policy evaluation in developing countries. Rev. Environ. Econ. Policy 20, prer019.

[12] Bauch SC, Birkenbach AM, Pattanayak SK, Sills EO 2015 Public health impacts of ecosystem change in the Brazilian Amazon. Proc. Natl Acad. Sci. USA 112, 7414–7419. (10.1073/pnas.1406495111) 26082548 PMC4475939

[13] Pattanayak SK, Pfaff A 2009 Behavior, environment, and health in developing countries: evaluation and valuation. Annu. Rev. Resour. Econ. 1, 183–217. (10.1146/annurev.resource.050708.144053)

[14] Wunder S 2015 Revisiting the concept of payments for environmental services. Ecol. Econ. 117, 234–243. (10.1016/j.ecolecon.2014.08.016)

[15] Sharma BP, Shyamsundar P 2015 Are community forestry institutions appropriate for implementing REDD+? Lessons from Nepal.

[16] Stanton T, Echavarria M, Hamilton K, Ott C 2010 State of watershed payments: an emerging marketplace. State of watershed payments: an emerging marketplace.

[17] Pattanayak SK 1997 Pricing ecological services provided by protected watersheds: micro-econometric applications in agrarian communities of Indonesia and the Philippines. Doctoral dissertation, Duke University, Durham, NC, USA.

[18] Chichilnisky G, Heal G 1998 Economic returns from the biosphere. Nature 12, 629–630. (10.1038/35481)

[19] Vincent JR, Ahmad I, Adnan N, Burwell WB III, Pattanayak SK, Tan-Soo JS, Thomas K 2016 Valuing water purification by forests: an analysis of Malaysian panel data. Environ. Resour. Econ. 64, 59–80. (10.1007/s10640-015-9934-9)

[20] Obidzinski K, Andriani R, Komarudin H, Andrianto A 2012 Environmental and social impacts of oil palm plantations and their implications for biofuel production in Indonesia. Ecol. Soc. 17, 25 (10.5751/ES-04775-170125)

[21] DeFries RS et al. 2012 Planetary opportunities: a social contract for global change science to contribute to a sustainable future. BioScience 62, 603–606. (10.1525/bio.2012.62.6.11)

[22] Pattanayak SK, Ross MT, Depro BM, Bauch SC, Timmins C, Wendland KJ, Alger K 2009 Climate change and conservation in Brazil: CGE evaluation of health and wealth impacts. BEJ Econ. Anal. Policy 9, 6 (10.2202/1935-1682.2096)

[23] Vincent JR 2016 Impact evaluation of forest conservation programs: benefit-cost analysis, without the economics. Environ. Resour. Econ. 63, 395–408. (10.1007/s10640-015-9896-y)

[24] Ravallion M 2009 Should the randomistas rule? Econ. Voice 6, 1–5.

[25] Dasgupta P 2001 Valuing objects and evaluating policies in imperfect economies. Econ. J. 111, 1–29. (10.1111/1468-0297.00617)

[26] Brownell KD, Roberto CA 2015 Strategic science with policy impact. Lancet 385, 2445–2446. (10.1016/S0140-6736(14)62397-7) 25703107

[27] Cash DW, Clark WC, Alcock F, Dickson NM, Eckley N, Guston DH, Jäger J, Mitchell RB 2003 Knowledge systems for sustainable development. Proc. Natl Acad. Sci. USA 100, 8086–8091. (10.1073/pnas.1231332100) 12777623 PMC166186

[28] Clark WC, van Kerkhoff L, Lebel L, Gallopin GC 2016 Crafting usable knowledge for sustainable development. Proc. Natl Acad. Sci. USA 113, 4570–4578. (10.1073/pnas.1601266113) 27091979 PMC4855559

